# Involvement of lncRNA TUG1 in HIV-1 Tat-Induced Astrocyte Senescence

**DOI:** 10.3390/ijms24054330

**Published:** 2023-02-22

**Authors:** Prakash P. Pillai, Muthukumar Kannan, Susmita Sil, Seema Singh, Annadurai Thangaraj, Ernest T. Chivero, Raghubendra Singh Dagur, Ashutosh Tripathi, Guoku Hu, Palsamy Periyasamy, Shilpa Buch

**Affiliations:** 1 Department of Pharmacology and Experimental Neuroscience, University of Nebraska Medical Center, Omaha, NE 68198, USA; 2 Division of Neurobiology, Department of Zoology, Faculty of Science, The Maharaja Sayajirao University of Baroda, Vadodara 390002, Gujarat, India; 3 Centre for Excellence in Nanobio Translational Research, Anna University, BIT Campus, Tiruchirappalli 600025, Tamil Nadu, India; 4 Department of Psychology, University of Nebraska Omaha, Omaha, NE 68182, USA; 5 Louis A. Faillace, MD, Department of Psychiatry and Behavioral Sciences at McGovern Medical School, The University of Texas Health Science Centre at Houston, Houston, TX 77030, USA

**Keywords:** aging, astrocyte senescence, HIV-1 Tat, lncRNA TUG1, proinflammatory cytokines

## Abstract

HIV-1 infection in the era of combined antiretroviral therapy has been associated with premature aging. Among the various features of HIV-1 associated neurocognitive disorders, astrocyte senescence has been surmised as a potential cause contributing to HIV-1-induced brain aging and neurocognitive impairments. Recently, lncRNAs have also been implicated to play essential roles in the onset of cellular senescence. Herein, using human primary astrocytes (HPAs), we investigated the role of lncRNA TUG1 in HIV-1 Tat-mediated onset of astrocyte senescence. We found that HPAs exposed to HIV-1 Tat resulted in significant upregulation of lncRNA TUG1 expression that was accompanied by elevated expression of p16 and p21, respectively. Additionally, HIV-1 Tat-exposed HPAs demonstrated increased expression of senescence-associated (SA) markers—SA-β-galactosidase (SA-β-gal) activity and SA-heterochromatin foci—cell-cycle arrest, and increased production of reactive oxygen species and proinflammatory cytokines. Intriguingly, gene silencing of lncRNA TUG1 in HPAs also reversed HIV-1 Tat-induced upregulation of p21, p16, SA-β gal activity, cellular activation, and proinflammatory cytokines. Furthermore, increased expression of astrocytic p16 and p21, lncRNA TUG1, and proinflammatory cytokines were observed in the prefrontal cortices of HIV-1 transgenic rats, thereby suggesting the occurrence of senescence activation in vivo. Overall, our data indicate that HIV-1 Tat-induced astrocyte senescence involves the lncRNA TUG1 and could serve as a potential therapeutic target for dampening accelerated aging associated with HIV-1/HIV-1 proteins.

## 1. Introduction

Although the advent of combination antiretroviral therapy (cART) has normalized the life expectancy of people living with HIV-1 (PLWH) to that of uninfected individuals, there is also an increased prevalence of HIV-1-associated neurocognitive disorders (HAND) and related comorbidities as these individuals live longer lifespans [1,2,3]. Adding further complexity to this is the preponderance of age-related mental health comorbidities of the elderly afflicting PLWH, ultimately manifesting as premature aging [4,5,6,7]. Studies have demonstrated the accumulation of senescent cells in the aging brain [8]. The contribution of HIV-1-associated mediators to the induction of cellular senescence has been demonstrated in mesenchymal stem cells [9], corneal endothelial cells [10], CD8+ T-cells [11], CD4+ T-cells [12], microglia [4,13], and astrocytes [14]. Additionally, life-long dependence on cART as well as dependence on illicit drugs such as methamphetamine have also been implicated in inducing astrocyte senescence [14,15].

Owing to the fact that despite cART, HIV-1 proteins such as transactivator of transcription (Tat) and gp120 continue to persist in tissues such as the brain and lymph nodes [16,17,18], we sought to understand the role of HIV-1 Tat in mediating astrocyte senescence. HIV-1 Tat is an early viral protein released by HIV-1-infected cells such as microglia, macrophages, and astrocytes and crosses the blood–brain barrier (BBB) from the periphery into the central nervous system (CNS), where it exerts is cytotoxicity directly by acting on glial and neuronal cells [19,20,21,22,23,24]. HIV-1 Tat-mediated cytotoxicity further leads to the release of soluble factors underlying neuroinflammation, oxidative stress, and excitotoxicity, ultimately resulting in neuronal damage [16,25].

Astrocytes, one of the most abundant glial cell populations in the CNS, perform key roles involving synapse formation and function, the release and uptake of neurotransmitters, the production of neurotrophic factors, the control of neuronal survival, which regulate BBB integrity and contribute to immunity within the CNS [14,26,27]. An impairment of astrocytes thus plays a significant role in the onset and progression of neurodegenerative diseases [28,29]. Even though HIV-1-infected astrocytes make up a small fraction of the infected cells within the CNS, these glial cells still represent a critical population that exhibits increased sensitivity to inflammatory triggers [30,31], ultimately resulting in neuronal impairment associated with HAND. In addition to active HIV-1 infection, viral proteins such as HIV-1 Tat and gp120 can also modulate astrocyte dysfunction and trigger inflammatory responses [32]. The accumulation of senescent astrocytes has also been demonstrated in Alzheimer’s disease [33] and is associated with exposure of the host to environmental factors leading to the development of Parkinson’s disease [34]. HIV-1 infection has been reported to induce macrophage and microglia senescence involving alterations in senescence-associated β-galactosidase (SA-β-gal) activity, p21 levels, and the production of cytokines such as IL6 and IL8, all of which contribute to HAND [4,13]. There is, however, a paucity of knowledge on the role of HIV-1 infection or HIV-1 proteins in driving astrocyte senescence leading to accelerated aging.

Recently, various epigenetic modifications, including DNA methylation, histone modifications, and dysregulated expression of noncoding RNAs that accumulate across the lifespan, have been implicated in predicting several age-related complications such as cellular senescence [35,36,37,38,39]. While there is evidence of accelerated aging based on epigenetic data in PLWH [38,40], the role of noncoding RNAs in the context of HIV-1 protein(s)-mediated cellular senescence remains elusive.

Long noncoding RNAs (lncRNAs) that are poorly conserved but abundant heterogeneous regulatory noncoding RNAs regulate gene expression at multiple levels, including transcription, RNA processing, translation, and post-translation. It has also been demonstrated that numerous lncRNAs mediate cellular senescence in various stages of the cell cycle by modulating senescence-associated pathways, such as p53/p21, pRB/p16, and p14 [41,42,43,44,45]. LncRNA Taurine Upregulated Gene 1 (TUG1) is one of the novel lncRNAs that is primarily expressed in retinal and brain tissues [46,47,48]. LncRNA TUG1, along with other lncRNAs, have been shown to regulate the cell cycle, which is specifically increased during aging [49]. LncRNA TUG1 has also been shown to disrupt the expression of the Homeobox (HOX) gene family, such as HOXB7, thereby leading to an aging phenotype [50]. LncRNA TUG1 is highly expressed in the human subependymal zone of the brain and is involved in age-related neurodegenerative diseases such as ischemic stroke and Huntington’s disease [49,51,52,53]. LncRNA TUG1 has an impact on tissue-specific aging such as intervertebral disk and age-related cataract involving the Wnt/β-catenin/caspase pathways [54,55]. The expression of upregulated lncRNA TUG1 has been shown to promote the proliferation of esophageal squamous cells and plays a role in promoting the proliferation of non-small-cell lung carcinoma [56]. Despite these diverse effects, the potential role of lncRNA TUG1 in astrocyte proliferation/senescence has not been examined to date.

In this study, we determined the role of the lncRNA TUG1 in the onset of HIV-1 Tat-induced astrocyte senescence. Our findings implicate the role of lncRNA TUG1 as a novel regulator of astrocyte senescence that could be targeted as a therapeutic option for ameliorating HIV-1 Tat-mediated accelerated aging in the context of HAND.

## 2. Results

### 2.1. HIV-1 Tat-Mediated Upregulation of Senescence-Associated Markers in Human Primary Astrocytes (HPAs)

**Figure 1:**
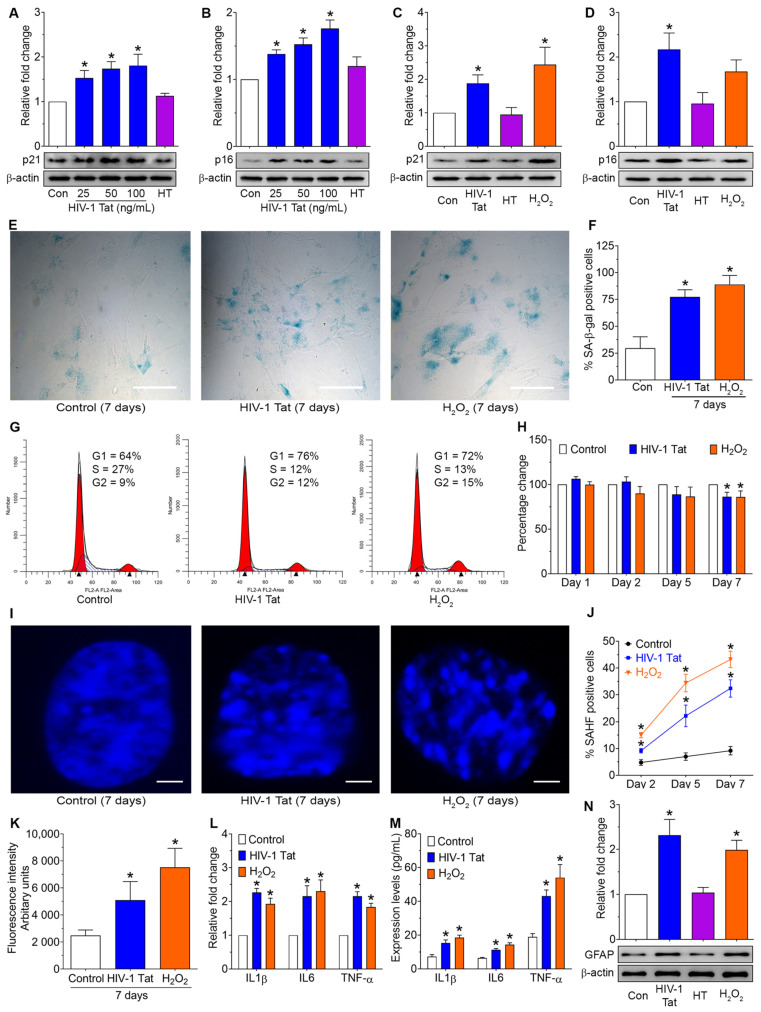
HIV-1 Tat increased the senescence-associated markers, ROS production, and proinflammatory cytokines and cellular activation in human primary astrocytes (HPAs). (**A**,**B**) Representative western blotting analysis showing the dose-dependent upregulation of p21 (**A**) and p16 (**B**) expression in HIV-1 Tat-exposed HPAs for 24 h. (**C**,**D**) Representative western blotting analysis showing the upregulation of p21 (**C**) and p16 (**D**) expression in HIV-1 Tat- (50 ng/mL) exposed HPAs for 7 days. β-actin was probed as a protein loading control for all experiments. (**E**,**F**) Representative images showing the cellular morphology and SA-β-gal (blue cells) staining (**E**) and quantification of SA-β-gal positive cells (**F**) on HIV-1 Tat-exposed HPAs for 7 days. Scale bar: 100 μm. (**G**) Flow cytometry analysis using PI staining showing the cell cycle analysis in HIV-1 Tat-exposed HPAs for 7 days. (**H**) Bar graph showing cell viability of control and HIV-1 Tat-exposed HPAs for 1, 2, 5, and 7 days. (**I**,**J**) Representative images showing the SAHFs in DAPI staining of HIV-1 Tat-exposed HPAs for 2, 5, and 7 days. Scale bar: 2 μm. (**K**) Representative DCFDA/H2DCFDA assay showing the quantification of the production of ROS in HIV-1 Tat-exposed HPAs for 7 days. (**L**,**M**) qPCR and ELISA data showing the mRNA and protein expression levels of proinflammatory cytokines, such as IL1β, IL6, and TNF-α in HIV-1 Tat-exposed HPAs for 7 days. GAPDH was used as a housekeeping control for qPCR (**N**) Representative western blotting analysis showing the upregulation of GFAP expression in HIV-1 Tat- (50 ng/mL) exposed HPAs for 7 days. β-actin was used as an internal control for all experiments. Data are mean ± SEM from 3 independent experiments. One-way ANOVA followed by Dunn’s post hoc test was used to determine the statistical significance of multiple groups. * *p* < 0.05 versus control. Con, Control. HT, Heat-inactivated HIV-1 Tat, served as a negative control. H_2_O_2_ (150 μM for 2 h) served as a positive control.

To determine whether exposure to HIV-1 Tat induces astrocyte senescence, HPAs were exposed to varying doses of HIV-1 Tat (25, 50, and 100 ng/mL) for 24 h, following which the expression levels of cellular senescence markers such as p21^Waf1^ and p16^Ink4a^ were determined using western blotting. As shown in Figure 1, exposure of HPAs to HIV-1 Tat significantly (*p* < 0.05) increased the expression levels of p21 (Figure 1A) and p16 (Figure 1B) in a dose-dependent manner. Chronic exposure of HPAs to HIV-1 Tat (50 ng/mL) for seven days (HIV-1 Tat was added to the cells daily at the same time) also significantly (*p* < 0.05) increased the expression levels of p16 (Figure 1C) and p21 (Figure 1D). The concentration of 50 ng/mL of HIV-1 Tat was thus chosen for the subsequent experiments. Previous reports have demonstrated the circulating levels of HIV-1 Tat in the cerebrospinal fluid of HIV-1-infected patients to range from 1 to 40 ng/mL [16,25,57], and this is speculated to be even higher, especially in the proximity of HIV-1-positive perivascular cells. We next performed the senescence-associated-β-galactosidase (SA-β-gal) staining in HPAs exposed to HIV-1 Tat (50 ng/mL; 7 days), and as shown in Figure 1E,F, the SA-β-gal staining intensity was notably elevated in HIV-1 Tat-exposed HPAs compared with control cells. The exposure of HPAs to HIV-1 Tat (50 ng/mL) also mediated cell cycle arrest at the G0/G1 phase as determined by flow cytometry. As shown in Figure 1G, exposure of HPAs to HIV-1 Tat increased the accumulation of cells in the G1 phase (from 60.76% to 72.83%) with a concomitant decrease in the S phase of cells (from 16.87% to 9.70%), thereby suggesting cell cycle arrest at the G0/G1 phase during astrocyte senescence induced by HIV-1 Tat. In these studies, exposure of cells to hydrogen peroxide (H_2_O_2_) served as a positive control. In addition, we also demonstrated that exposure of HPAs to HIV-1 Tat (50 ng/mL; 7 days) failed to induce cell death in our experimental paradigm (Figure 1H). HIV-1 Tat-mediated astrocyte senescence was further characterized by increased formation of senescence-associated heterochromatin foci (SAHF) as shown by punctated DAPI staining (Figure 1I). Moreover, the percentage of cells containing SAHF-positive foci was significantly increased from day 2 to day 7 in HIV-1 Tat-exposed HPAs compared with the control group (Figure 1J). Next, we sought to examine whether exposure of HPAs to HIV-1 Tat induced the generation of ROS production using a 2′,7′-dichlorodihydrofluorescein diacetate (H_2_DCF DA) fluorescence assay. As shown in Figure 1K, exposure of HPAs to HIV-1 Tat for 7 days increased ROS production compared with control cells. H_2_O_2_ was used as a positive control (Figure 1K).

Next, we determined the senescence-associated secretory phenotypes (SASPs) in HPAs exposed to HIV-1 Tat (50 ng/mL; for 7 days) by determining the expression levels of proinflammatory cytokines such as TNF and IL6 mRNAs using both qPCR and ELISA. As shown in Figure 1L,M, HPAs exposed to HIV-1 Tat demonstrated a significant (*p* < 0.05) increase in the mRNA and protein expression of proinflammatory cytokines, such as IL1β, IL6, and TNF-α compared with control cells. To further confirm whether exposure of HPAs to HIV-1 Tat could impact cellular activation, HPAs were exposed to HIV-1 Tat (50 ng/mL; 7 days) and assessed for the expression of glial fibrillary acidic protein (GFAP) using western blotting. As shown in Figure 1N, HPAs exposed to HIV-1 Tat demonstrated significantly (*p* < 0.05) increased expression of GFAP compared with control cells.

### 2.2. HIV-1 Tat Upregulates the Expression of lncRNA TUG1 in HPAs

To understand the mechanisms underlying HIV-1 Tat-induced astrocyte senescence in vitro, we next sought to assess the involvement of lncRNAs in cellular senescence and aging.

**Figure 2:**
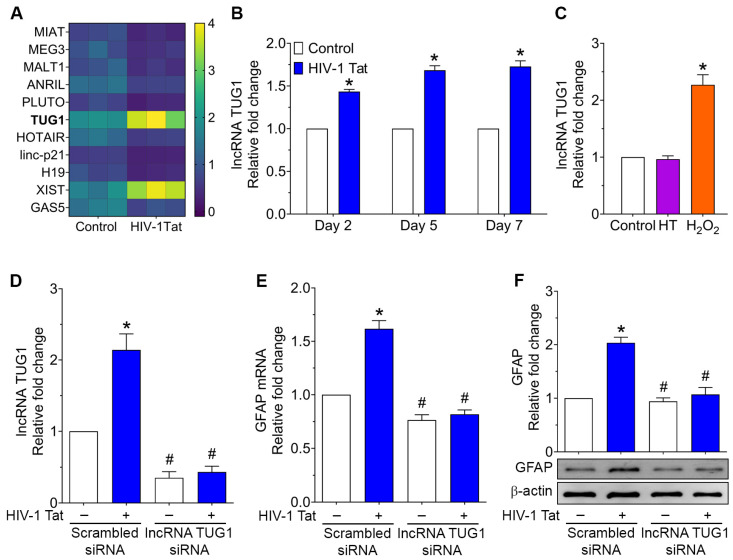
HIV-1 Tat increased lncRNA TUG1 expression and cellular activation in HPAs. (**A**) Heatmap of custom lncRNA qPCR showing the differential expression of various senescence-associated lncRNAs in HIV-1 Tat-exposed HPAs. (**B**) qPCR showing the expression of time-dependent upregulation of lncRNA TUG1 in HIV-1 Tat-exposed HPAs for 2, 5, and 7 days. GAPDH was used as a housekeeping control. (**C**) qPCR showing the expression of lncRNA TUG1 in heat-inactivated HIV-1 Tat or H_2_O_2_ (150 μM for 2 h) exposed HPAs. GAPDH was used as a housekeeping control. (**D**) qPCR showing the silencing efficiency of lncRNA TUG1 in HPAs transfected with siRNAs of scrambled and lncRNA TUG1 followed by HIV-1 Tat exposure. GAPDH was used as a housekeeping control. Representative qPCR (**E**) and western blotting (**F**) showing the expression levels of GFAP in HPAs transfected with siRNAs of scrambled and lncRNA TUG1 followed by HIV-1 Tat exposure. β-actin was used as an internal control for western blotting. GAPDH was used as a housekeeping control for qPCR. Data are mean ± SEM from 3 independent experiments. One-way ANOV followed by Dunn’s post hoc test was used to determine the statistical significance of multiple groups. * *p* < 0.05 versus control; # *p* < 0.05 versus HIV-1 Tat.

Herein, we monitored the expression levels of known senescence-associated lncRNAs, such as MIAT, MEG3, MALAT1, ANRIL, PLUTO, HOTAIR, H19, lncRNA p21, XIST, GAS5, and TUG1 in HIV-1 Tat-induced astrocyte senescence. As shown in Figure 2A, HPAs exposed to HIV-1 Tat (50 ng/mL) significantly (*p* < 0.05) downregulated the expression of all the lncRNAs except for lncRNA TUG1, which was significantly upregulated compared with control cells. Since lncRNA TUG1 has been shown to play a role in age-related neurodegenerative diseases and cellular senescence, we further sought to determine the expression levels of lncRNA TUG1 in HIV-1 Tat- (50 ng/mL for 2, 5, and 7 days) exposed HPAs in vitro. As shown in Figure 2B, we found a significant upregulation of lncRNA TUG1 in HPAs exposed to HIV-1 Tat. In addition, the expression of lncRNA TUG1 was significantly (*p* < 0.05) upregulated in HPAs exposed to H_2_O_2_ with no significant changes in HPAs exposed to heat-inactivated HIV-1 Tat (Figure 2C). We also wanted to determine the contribution of upregulated lncRNA TUG1 in HIV-1 Tat-induced astrocyte activation. For this, we used the gene silencing approach by knocking down lncRNA TUG1 in HPAs, followed by exposing cells to HIV-1 Tat (50 ng/mL; 2 days). Figure 2D demonstrates the gene silencing efficiency. We next sought to determine the astrocyte activation in this experimental setup, and as shown in Figure 2E,F, in HPAs silenced for the lncRNA TUG1 expression, there was a significant (*p* < 0.05) abrogation of HIV-1 Tat-mediated upregulation of GFAP mRNA (Figure 2E) and protein expression (Figure 2F) within 2 days.

### 2.3. Gene Silencing of lncRNA TUG1 Prevents HIV-1 Tat-Induced Cellular Senescence in HPAs

**Figure 3:**
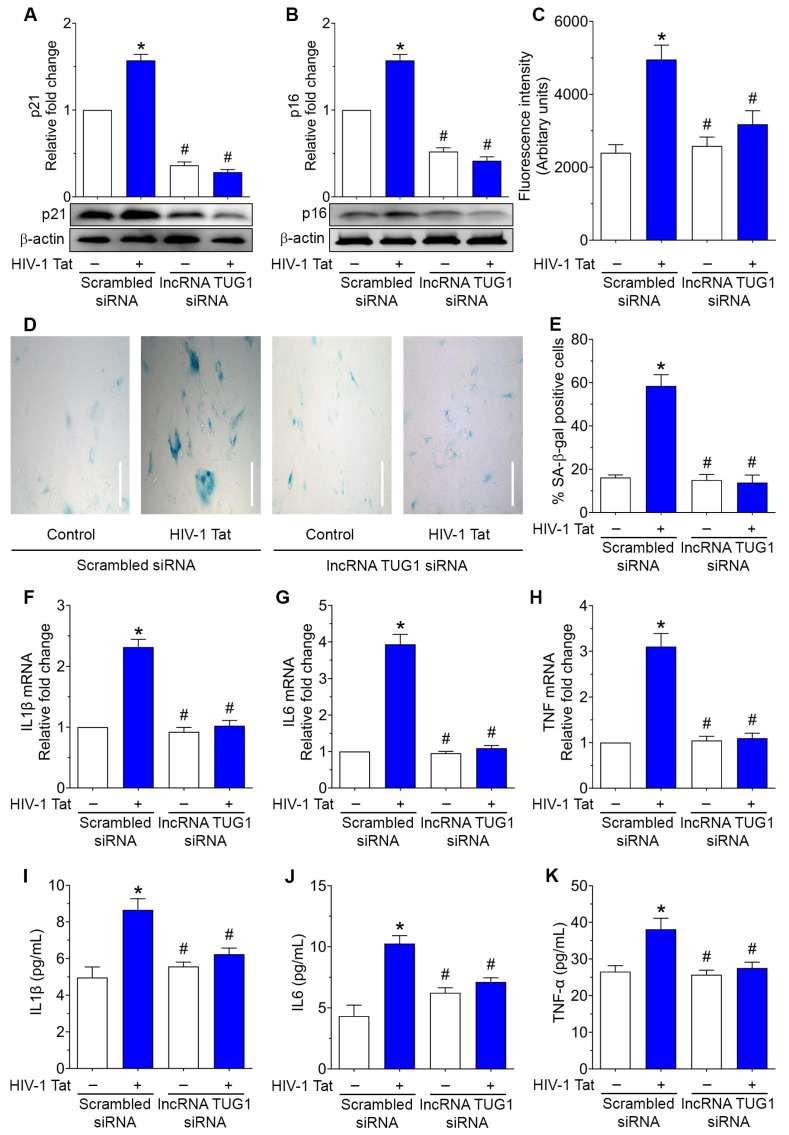
Gene silencing of lncRNA TUG1 prevented HIV-1 Tat-induced cellular senescence in HPAs. Representative western blotting showing the expression levels of p21 (**A**) and p16 (**B**) in HPAs transfected with siRNAs of scrambled and lncRNA TUG1 followed by HIV-1 Tat exposure. β-actin was used as an internal control for all experiments. (**C**) Bar graph showing the ROS production levels in HPAs transfected with siRNAs of scrambled and lncRNA TUG1 followed by HIV-1 Tat exposure. Representative SA-β-gal staining (**D**) and quantification (**E**) in HPAs transfected with siRNAs of scrambled and lncRNA TUG1 followed by HIV-1 Tat exposure. Scale bar: 100 μm. qPCR showing the mRNA expression of proinflammatory cytokines, such as IL1β (**F**), IL6 (**G**), and TNF (**H**) in HPAs transfected with siRNAs of scrambled and lncRNA TUG1 followed by HIV-1 Tat exposure. GAPDH was used as a housekeeping control. ELISA quantification showing the protein levels of proinflammatory cytokines, such as IL1β (**I**), IL6 (**J**), and TNF (**K**) in HPAs transfected with siRNAs of scrambled and lncRNA TUG1 followed by HIV-1 Tat exposure. Data are mean ± SEM from 6 independent experiments. One-way ANOVA followed by Dunn’s post hoc test was used to determine the statistical significance of multiple groups. * *p* < 0.05 versus control; # *p* < 0.05 versus HIV-1 Tat.

Next, we sought to determine the contribution of lncRNA TUG1 in HIV-1 Tat-induced astrocyte senescence and increased expression of proinflammatory cytokines in lncRNA TUG1 silenced in HPAs exposed to HIV-1 Tat (50 ng/mL; 2 days). As shown in Figure 3A,B, transfection of HPAs with lncRNA TUG1 siRNA, but not the scrambled siRNA, significantly (*p* < 0.05) downregulated HIV-1 Tat-induced expression of the senescence markers such as p21 (Figure 3A) and p16 (Figure 3B). As expected, in HPAs transfected with scrambled siRNA, HIV-1 Tat exposure resulted in increased production of ROS and increased numbers of SA-β-gal activity compared with the control cells, whereas, silencing of lncRNA TUG1 in HPAs resulted in a significant (*p* < 0.05) reduction in both ROS levels (Figure 3C) and the number of SA-β-gal-positive cells (Figure 3D,E). Furthermore, gene silencing of lncRNA TUG1 in HPAs also significantly (*p* < 0.05) abrogated the expression levels of proinflammatory cytokines, such as IL1β (Figure 3F), IL6 (Figure 3G) and TNFα (Figure 3H) mRNAs compared with control cells. Similarly, and as expected, the protein expression levels of the proinflammatory cytokines (Figure 3I–K) were also normalized in lncRNA TUG1-silenced, HIV-1 Tat-exposed HPAs.

### 2.4. Validation of Senescence-Associated Markers in HIV-1 Transgenic (Tg) Rats

**Figure 4:**
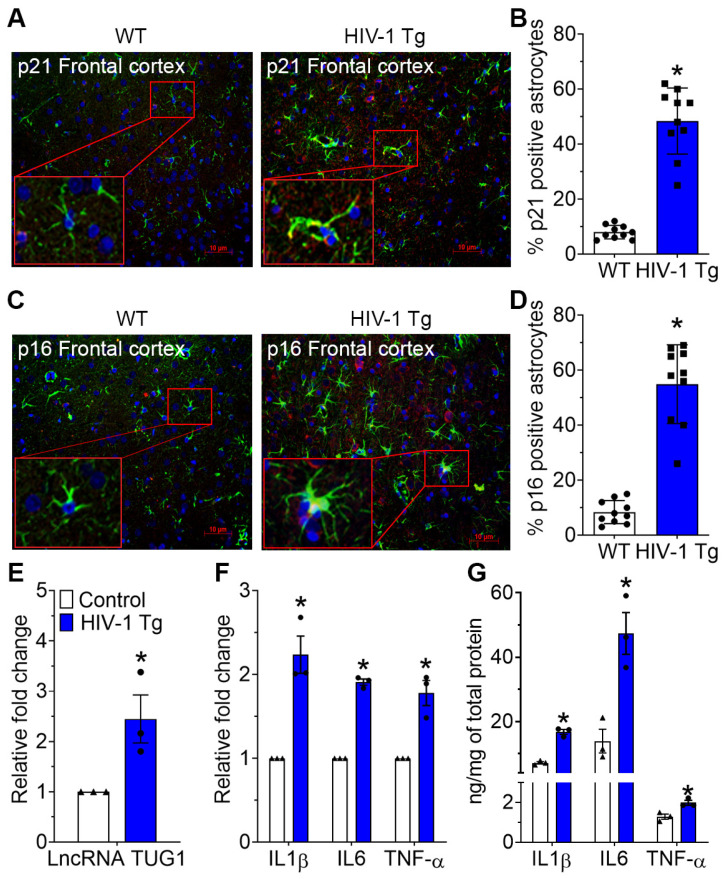
HIV-1 Tg rats showed increased astrocytic senescence-associated markers. Representative immunofluorescence images showing the colocalization of p21 (**A**,**B**) and p16 (**C**,**D**) with GFAP in the prefrontal cortices of wild-type and HIV-1 Tg rats. Scale bar: 10 μm. qPCR showing the expression of lncRNA TUG1 (**E**) in the prefrontal cortices of wild-type and HIV-1 Tg rats. qPCR (**F**) and ELISA (**G**) showing the mRNA and protein expression of proinflammatory cytokines, such as IL1β, IL6, and TNFα in the prefrontal cortices of wild-type and HIV-1 Tg rats. GAPDH was used as a housekeeping control for qPCR. Data are mean ± SEM. N = 3/group. A Student’s t-test was used to determine statistical significance. * *p* < 0.05 versus control.

Having shown HIV-1 Tat-mediated astrocyte senescence in vitro, we next sought to validate our findings in vivo using HIV-1 Tg rats. For this, 15-month-old HIV-1 Tg rats and age-matched wild-type rats were assessed for the expression of p21 and p16 in the prefrontal cortex. As shown in Figure 4A,B, we performed immunostaining for the expression of p21 and p16 that colocalize with GFAP in these groups of rats and found significant colocalization of p21 (Figure 4A,B) and p16 with GFAP (Figure 4C,D) in the prefrontal cortices of HIV-1 Tg rats compared to that of wild-type rats. Additionally, the expression of lncRNA TUG1 as well as the proinflammatory cytokines, such as IL1β, IL6 and TNFα (both mRNA and protein levels) were monitored in the prefrontal cortices of HIV-1 Tg and wild-type rats using qPCR and ELISA. As expected, the expression of lncRNA TUG1 (Figure 4E) and the mRNA and protein expression levels of proinflammatory cytokines (Figure 4F,G) were significantly (*p* < 0.05) increased in the prefrontal cortices of HIV-1 Tg rats compared with the wild-type rats.

## 3. Discussion

With the availability of effective cART, HIV-1 has transformed from a death sentence to a manageable chronic disease, with many individuals living longer and healthier lives. In fact, with the right treatment regimen and care, the life expectancy of those infected with HIV-1 can be comparable to uninfected healthy individuals. However, there is ample evidence implicating that PLWH have a higher risk of developing certain age-related illnesses [2,58]. As stated above, chronic infection with HIV-1, coupled with early initiation and dependence on cART and drug abuse, results in a slow-smoldering inflammatory milieu in the brain that accumulates over time, culminating into premature aging and neurodegeneration. In the HIV-1-infected, cART-treated, drug-abusing population, these neurodegenerative, cognitive impairments are seen at a much younger age compared with normal healthy (uninfected and drug-naïve) counterparts [59,60]. Some of the likely mediators contributing to HIV-1-associated premature aging include residual viral proteins such as HIV Tat and gp120 and long-term toxicity of the antiretrovirals, among others [61,62,63].

In the current study, we demonstrate that exposure of HPAs to HIV-1 Tat upregulated the expression of lncRNA TUG1, which, in turn, correlated with increased SA-β-gal activity, p16, and p21 levels, the formation of SAHF, and the acquisition of a SASP in astrocytes. Interestingly, our findings also demonstrated that gene silencing of lncRNA TUG1 was able to reverse HIV-1 Tat-induced astrocyte senescence, thereby suggesting a possible involvement of the lncRNA TUG1/p16/p21 axis in HIV-1 Tat-mediated astrocyte senescence. Our previously published reports using HIV-1 Tg rats and HPAs underpinned the role of the HIV-1 Tat protein in inducing a set of microRNAs that played a critical role in astrogliosis with implications in aging [64]. Our findings are comparable to earlier studies implicating the role of HIV-1 infection or the HIV-1 Tat protein and other inducers, such as drugs of abuse (methamphetamine) or antiviral drugs, as contributors of astrocyte senescence [14,15] with the acquisition of classical senescence markers along with the increased expression of proinflammatory cytokines and oxidative stress.

It is well recognized that only cells with stable cell cycle arrest are considered senescent and that the pathways underlying this process are driven by cyclin-dependent kinase inhibitors such as p21 and p16 [65]. Our study also demonstrated that increased expression of p21 and p16 proteins in HPAs exposed to HIV-1 Tat plays a critical role in the induction of stable cell cycle arrest in these cells. Additionally, we also demonstrate that in the brains of the HIV-1 Tg rats, there is an increased proportion of astrocytes expressing p21 and p16 in the prefrontal cortices compared with the wild-type rats, thus suggesting increased activation of senescence and cell cycle dysregulation. These observations are consistent with current reports showing that there is an increased expression of p21 and p16 in post-mortem brain tissues of Amyotrophic Lateral Sclerosis/Motor Neurone Disease [66]. Similar observations were also reported in the brains of individuals with Alzheimer’s disease [33]. We also showed an increased number of astrocytes in the G0/G1 phase of the cell cycle following HIV-1 Tat exposure. These observations further confirm impaired cell-cycle regulation in the HIV-1 Tat-exposed HPAs.

Senescent cells are morphologically characterized by an enlarged size and flattened shape compared with the non-senescent control cells. The senescent cells also exhibit elevated levels of ROS, increased lysosomal contents, and compromised lysosomal activity, ultimately resulting in increased levels of β-galactosidase [67,68]. Data from the current in vitro astrocyte senescence model confirm these metabolic and morphological changes. A characteristic feature of senescent cells is extensive chromatin reorganization resulting in the formation of SAHF [69,70]; nuclei of senescent cells typically contain 30–50 bright, punctate, DNA-stained, dense foci that can be readily distinguished from chromatin in normal cells. The SAHFs play a role in sequestering proliferation-promoting genes that are required for the progression through the S-phase of the cell cycle [71]. As shown in our study, there is increased DAPI puncta representing SAHFs in senescent astrocytes by day 5 in vitro, which reached more than 50% by day 7 in vitro. Many senescent cells acquire a SASP that comprises a highly complex mixture of secreted cytokines, chemokines, growth factors, and proteases. The chronic inflammation induced by the sustained presence of senescent astrocytes leads to tissue damage and degeneration in the aged brain. Here, we show increased mRNA expression of proinflammatory cytokines following HIV-1 Tat exposure. IL6 is one of the prominent cytokines of the SASP, and our data also indicate a significant increase in the gene expression levels of IL6. This increased proinflammatory cytokine gene expression confirms the involvement of immune activation in astrocyte senescence. Our data are in line with the earlier reports wherein astrocytes were demonstrated to be an important source of the generation of proinflammatory mediators in response to HIV-1/HIV-1 proteins [19,30,72,73]. The astrocytes are not only the producers of neuroinflammation but are also known to be highly susceptible to both the viral proteins and cellular toxins released from HIV-1-infected macrophages/microglia. Upon activation, the astrocytes undergo astrogliosis that is characterized by cellular proliferation and further release of toxic mediators. There are also reports suggesting age-related upregulation of GFAP expression as well as astrogliosis in different brain regions [74,75,76,77]. Here, we show increased gene expression of GFAP mRNA in astrocytes following continuous exposure to HIV-1 Tat, suggesting the involvement of glial activation—one of the key phenotypic features of the senescing astrocytes.

Recent advances in high-throughput sequencing and RNA profiling technologies have facilitated the identification of an enormous number of lncRNAs and their critical roles in diverse biological processes including cellular senescence and aging [78,79]. In addition, emerging evidence indicates that aberrant expression of lncRNAs is associated with neuronal aging, cellular senescence, and tumors, suggesting their involvement in regulating cell cycle events [80,81]. lncRNA TUG1 has been linked with promoting cell proliferation [82,83,84,85], suggesting tissue- and cell-type-specific lncRNA functions. In particular, a study conducted by Zhang et al. (2014) showed that lncRNA TUG1 is a direct target of the tumor suppressor p53 and has a role in repressing specific genes involved in cell-cycle regulation [56]. In the present study, we have determined the expression levels of lncRNAs that are known to be associated with cellular senescence, including lncRNA TUG1 in HPAs exposed to HIV-1 Tat. Our results demonstrate that out of several lncRNAs that were dysregulated, lncRNA TUG1 expression was significantly upregulated in HPAs exposed to HIV-1 Tat. Similar to findings in the present study, others have also reported downregulated lncRNA TUG1 expression in glioma and non-small-cell lung cancer [54,56].

**Figure 5:**
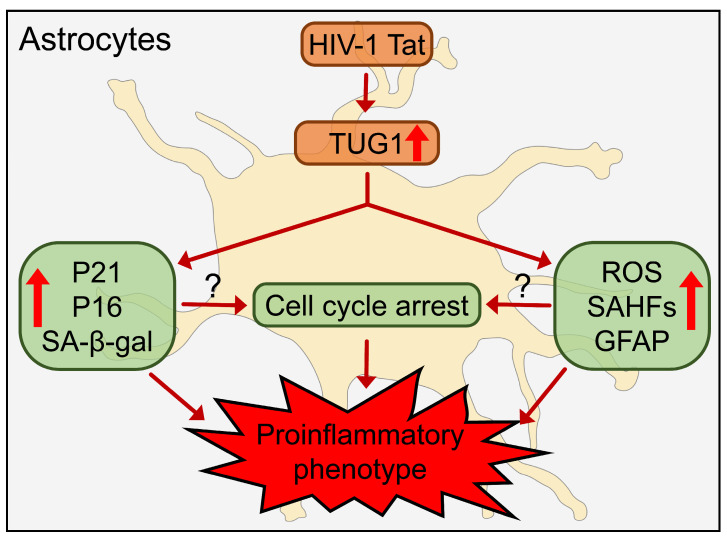
Schematic diagram depicting lncRNA TUG1-mediated astrocyte senescence in the context of HIV-1 Tat.

In summary, HIV-1 Tat increases SA-β-gal activity, p21 and p16 expression, cell cycle arrest, ROS, the formation of SAHFs, and the expression of proinflammatory cytokines and lncRNA TUG1 in HPAs. Intriguingly, silencing of lncRNA TUG1 retards most of these detrimental effects of HIV-1 Tat, such as the appearance of the proinflammatory phenotype as well as the increase in p21 and p16 expression (Figure 5).

Our findings linking lncRNA TUG1 to astrocyte senescence mediated by HIV-1 Tat is unique and previously not reported. Overall, our findings enrich the understanding of how lncRNA TUG1 may potentially drive cellular senescence in HPAs and initiate the accelerated aging process. These observations altogether suggest that lncRNA TUG1 could be considered as a therapeutic target to control accelerated or premature aging in PLWH.

## 4. Materials and Methods

### 4.1. Reagents

Endotoxin-free recombinant HIV-1 Tat-101 (1032–10) was purchased from ImmunoDX, LLC (Woburn, MA, USA). P16-INK4A (Cat No. 10883-1-AP) antibody was purchased from Proteintech Group, Inc. (Rosemont, IL, USA). P21 antibody (Cat No. ab109199) and GFAP (Cat No. ab18258) were purchased from Abcam, Boston, MA, USA. β-Actin (Cat No. sc-47778 HRP) antibody was purchased from Santa Cruz Biotechnology, Inc., Dallas, TX, USA. Senescence β-galactosidase staining kit (Cat No. 9860S) was purchased from Cell Signaling Technology, Inc. (Danvers, MA, USA). Hydrogen Peroxide (Cat No. H325-500) was purchased from Thermo Fisher Scientific, Inc. (Pittsburgh, PA, USA). Peroxidase-AffiniPure Goat Anti-Rabbit IgG (H + L) (Cat No. 111–035-003) and Peroxidase-conjugated AffiniPure Goat Anti-Mouse IgG (H + L) (Cat No. 115–035-003) was purchased from Jackson ImmunoResearch Inc. (West Grove, PA, USA). Image-IT™ LIVE Green Reactive Oxygen Species Detection Kit (Cat No. I36007), Lipofectamine™ RNAiMAX transfection reagent (Cat No. 13778150), Opti-MEM^®^ I Reduced Serum Media (Cat No. 31985070), goat anti-rabbit IgG (H + L) cross-adsorbed secondary antibody, Alexa Fluor 488 (Cat No. A-11008), goat anti-mouse IgG (H + L) cross-adsorbed secondary antibody, Alexa Fluor 594 (Cat No. A-11005), and ProLong^®^ Gold Antifade Mountant with DAPI (Cat No. P36935) were purchased from ThermoFisher Scientific, Inc. (Pittsburgh, PA, USA).

### 4.2. Animals

HIV-1 Tg rats (Harlon, Inc., Indianapolis, IN, USA; 15-month-old male rats) and age- and background-matched controls (F344) were used in this study (N = 3/group). All animal procedures were performed according to the protocols approved by the Institutional Animal Care and Use Committee (IACUC) of the University of Nebraska Medical Center (20-057-07-FC; Approved on 17 August 2020). Animals were euthanized and the prefrontal cortex was dissected and immediately snap-frozen and stored at −80 °C until used for various assays.

### 4.3. HPA Culture

HPAs were derived from the single-cell isolation process of fetal brain tissues. Briefly, human fetal brain tissues were collected from electively aborted specimens (gestational age 12–16 weeks) after the abortion procedure was completed in collaboration with the Birth Defects Research laboratory at the University of Washington. The protocol complies with all applicable state and federal requirements. All individuals provided informed consent using an Institutional Review Board-approved consent form at the University of Washington. For experiments, HPAs were cultured and appropriately treated in 6-well plates.

### 4.4. siRNA Transfection

HPAs with 60–80% confluent (0.8–1.0 × 10^6^ cells/well) in a 6-well plate for about 24 h after seeding, were treated with both lncRNA TUG1 siRNA (Sense: 5′-GGCCAACUUUGACAAAUCUUU-3′; Antisense: 5′-AGAUUUGUCAAAGUUGGCCUU-3′) and scrambled control siRNA (Sense: 5′-UUCUCCGAACGUGUCACGUUU-3′; Antisense: 5′-ACGUGACACGUUCGGAGAAUU-3′), as per standard protocols and reported previously [86]. Briefly, HPAs were transfected with targeted siRNA (20 pM) mixed with 2 μL of Lipofectamine™ RNAiMAX transfection reagent diluted in 100 μL of Opti-MEM^®^ I Reduced Serum Media. The resulting siRNA–lipid complexes were added to the HPAs and incubated for 6 h. Next, the medium was changed to 10% FBS-containing DMEM for 20 h incubation. The transfected HPAs were then ready for further experiments.

### 4.5. Flow Cytometric Analysis

Cell-cycle status in the HPAs was determined by measuring nuclear DNA content. The cells were collected on days 2, 5, and 7 after treatment with HIV-1 Tat, centrifuged, and washed twice with ice-cold PBS. The cells were then fixed in 70% ethanol at 4 °C for 24 h followed staining by propidium iodide (50 µg/mL) and RNAse A (100 μg/mL). The samples were analyzed using a FAC Calibur flow cytometer (BD Bioscience, San Diego, CA, USA), and the flow cytometry data were analyzed using FlowJo™ v10.6 software (TreeStar Inc., Ashland, OR, USA).

### 4.6. SA-β-Gal Staining

SA-β-gal staining was performed in HPAs treated with HIV-1 Tat for 7 days using the Senescence-β-galactosidase staining kit (Cell Signaling Technology, Inc., Danvers, MA, USA, Cat No. 9860S) as per the manufacturer’s instructions.

### 4.7. ROS Detection

ROS were detected in live cells using the Image-iT™ LIVE Green ROS Detection Kit according to the manufacturer’s instructions. Briefly, cells were seeded in 24-well plates followed by exposure to HIV-1 Tat, H_2_O_2_, or left untreated for up to 7 days. The cells were subsequently labeled with carboxy-H_2_DCFDA working solution (25 µM, 30 min at 37 °C), washed, mounted in the warm buffer, and imaged immediately on a Zeiss Observer using a Z1 inverted microscope (Carl Zeiss Microscopy, LLC. White Plains, NY, USA).

### 4.8. Assay for Senescence-Associated Heterochromatin Foci (SAHF)

HPAs treated with HIV-1 Tat or indicated chemicals for up to 7 days were subjected to SAHF quantification. SAHFs were measured by counting DAPI puncta following DAPI staining [69]. Briefly, control and treated HPAs were washed 3 times with ice-cold PBS and fixed with 4% PFA for 30 min. The fixed cells were then washed thrice with PBS and stained with DAPI (1 μg/mL; 10 min). After washing with PBS, the heterochromatin foci were imaged using a Z1 inverted microscope, and SAHF-positive cells were counted manually by independent blinded assessors.

### 4.9. MTT Cell Viability Assay

Cell viability was assessed using the MTT assay as per standard protocols and reported previously [87,88]. Briefly, HPAs were cultured in a 96-well plate (1 × 10^4^ cells per well) for 24 h followed by exposure either to HIV-1 Tat, heat-inactivated HIV-1 Tat, or H_2_O_2_ for an additional 24 h and incubated with 20 μL MTT tetrazolium salt (5 mg/mL) dissolved in Hank’s balanced salt solution added to each well and incubated in a CO_2_ incubator for 4 h. Finally, the medium was suctioned from each well, and 200 μL of dimethyl sulfoxide was added to disperse the formazan crystals. The absorbance of each well was obtained using a plate counter at the test and reference wavelengths of 570 and 630 nm, respectively.

### 4.10. Immunohistochemistry

Formalin-fixed, paraffin-embedded brain tissue sections of wild-type and HIV-1 Tg rats were baked at 60 °C overnight, followed by deparaffinization, rehydration, and antigen retrieval using the standard protocol. Then the slides were incubated with a blocking buffer containing 10% normal goat serum for 1 h at room temperature followed by the addition of primary antibodies, such as p21 (1:400), p16 (1:100), and GFAP (1:500), and incubated overnight at 4 °C. The next day, the sections were washed, followed by incubation with Alexa Fluor 488 goat anti-rabbit (1:500) and Alexa Fluor 555 goat anti-mouse (1:500), respectively, at room temperature for 2 h. The slides were then mounted, and fluorescent images were obtained using a Z1 inverted microscope (Carl Zeiss Microscopy, LLC, White Plains, NY, USA) for the colocalization analysis.

### 4.11. Western Blotting

Treated cells were lysed using the RIPA buffer (Cell Signaling Technology, Inc., Danvers, MA, USA, Cat No. 9806) and quantified using Pierce™ BCA Protein Assay Kit (ThermoFisher Scientific, Inc., Pittsburgh, PA, USA, Cat No. 23227). Equal amounts of proteins were electrophoresed and transferred on a polyvinylidene fluoride membrane (Millipore, Danvers, MA, USA, Cat No. IPVH00010). The membranes were blocked with 5% non-fat dry milk (in 1X TTBS buffer) and then probed overnight at 4 °C with primary antibodies, including p21 (1:2000), p16 (1:2000), GFAP (1:500), and β-actin (1:4000). After washing, appropriate secondary antibodies were added (1:5000) for 1 h at room temperature. Next, the blots were imaged and the data analyzed using ImageJ software [89].

### 4.12. Real-Time PCR

Real-Time PCR experiments were performed as per the standard protocol as described elsewhere. In brief, total RNA was isolated using Quick-RNA™ MiniPrep Plus (Zymo Research, Orange, CA, USA, Cat No. R1058) and reverse-transcribed using Verso cDNA synthesis kit (ThermoFisher Scientific, Inc., Pittsburgh, PA, USA, Cat No. AB1453B), as per the manufacturer’s protocols. Comparative real-time PCR was performed using RT^2^ SYBR Green ROX qPCR Mastermix (Qiagen, Germantown, MD, USA, Cat No. 330523) in an Applied Biosystems^®^ QuantStudio™ 3 Real-Time PCR System. The primers used in this study are shown in Table 1. Each PCR reaction was carried out in triplicate, and three independent experiments were run. GAPDH was used as a housekeeping control for the normalization, and the fold change in expression was obtained by the 2^−ΔΔCT^ method.

### 4.13. Statistical Analysis

Data are presented as mean  ±  SEM. For comparison between each group, Student’s t-test or one-way analysis of variance (ANOVA) with Dunn’s post hoc test was used. GraphPad Prism version 6.01 (San Diego, CA, USA) was used for statistical analysis and the preparation of bar graphs. A probability less than 0.05 was considered statistically significant.

## Figures and Tables

**Table 1:** List of primers used in this study.

Species	Gene	Forward	Reverse
Human	*GFAP*	5′-ATGGAGCTCAATGACCGCTTT-3′	5′-CGCCTTGTTTTGCTGTTCCA-3′
	*TNF*	5′-CAGCCTCTTCTCCTTCCTGAT-3′	5′-GCCAGAGGGCTGATTAGAGA-3′
	*IL1β*	5′-TACCTGTCCTGCGTGTTGAA-3′	5′-TCTTTGGGTAATTTTTGGGATCT-3′
	*IL6*	5′-CTGCAGCCACTGGTTCTGT-3′	5′-GGCACCCAGCACAATGAA-3′
	*GAPDH*	5′-TGCACCACCAACTGCTTAGC-3′	5′-ATGCCAGTGAGCTTCCCGTT-3′
	*TUG1*	5′-TGTCTCCATGCCTCAGATCTC-3′	5′-CAGCAGAGCCAGATTTGTCA-3′
	*GAS5*	5′-TATGGTGCTGGGTGCAGATG-3′	5′-ACGTTACCAGGAGCAGAACCAT-3′
	*XIST*	5′-CCCATTGAAGATACCACGCTG-3′	5′-ATCTCCACCTAGGGATCGTCAA-3′
	*MIAT*	5′-GGGAAATCTCTGGGACGTGA-3′	5′-GGAAAGACCCGCTTCATTGA-3′
	*MEG3*	5′-TGCCCATCTACACCTCACGA-3′	5′-GCATAGCAAAGGTCAGGGCTTA-3′
	*MALAT1*	5′-TGTGAGCACTTTCAGGAGAGC-3′	5′-TGCTTGGGAAATCTTAGAAACG-3′
	*ANRIL*	5′-TGCTTACCTAGTGCCAGATGCT-3′	5′-AATCCCAGCCAATTACCAGC-3′
	*HOTAIR*	5′-CTGTTACACGCCTCTCCAAGA-3′	5′-CAGGGTCCCACTGCATAATC-3′
	*PLUTO*	5′-GCTGGTGGCTGGAGAAACAT-3′	5′-AAAGAGTGGGCGTGAGCAA-3′
	*LincRNA p21*	5′-AGAAGCCTCCTTTCATCGGTTT-3′	5′-TCCTCCTTCAGCTCGGGTTA-3′
	*H19*	5′-TGGAGTCTGGCAGGAGTGAT-3′	5′-TGCCACGTCCTGTAACCAA-3′
Rat	*TUG1*	5′-AGAGGCAACAACTCACCCAG-3′	5′-GCACGGGACGTAGTTCACTT-3′
	*TNF*	5′-AAATGGGCTCCCTCTCATCAGTTC-3′	5′-TCCGCTTGGTGGTTTGCTACGAC-3′
	*IL1β*	5′-CACCTCTCAAGCAGAGCACAG-3′	5′-GGGTTCCATGGTGAAGTCAAC-3′
	*IL6*	5′-TCCTACCCCAACTTCCAATGCTC-3′	5′-TTGGATGGTCTTGGTCCTTAGCC-3′
	*GAPDH*	5′-GTATCGGACGCCTGGTTACC-3′	5′-CGCTCCTGGAAGATGGTGATGG-3′

## Data Availability

The data that support the findings of this study are available from the corresponding author upon reasonable request.
